# Supplementary material for: Learning curve and surgical outcome of robotic assisted colorectal surgery with ERAS program
Source: Sci Rep. 2022 Nov 29;12:20566. doi: 10.1038/s41598-022-24665-w (PMC9709162; doi:10.1038/s41598-022-24665-w)
Supplement: Supplementary file 2 — Supplementary Information 2. [file 41598_2022_24665_MOESM2_ESM.zip › RAL Raw data-Ñ[▒K/090/OP_ñw╝╨░O▒Kñσ.pdf]

正式摘要(電子簽章：000000218421881)

請選擇...reporter

臺中榮民總醫院

Operation Note

[redacted]

- 手術日期：2021/06/05
- 手術醫師：蔣鋒帆，CR 楊曙榮，R 陳瑨夫
- Anesthesia：GE
- Position：Lithotomy
- Aseptic agents：Soap，Betadine Alcohol，Betadine Aqua

Diagnosis

- Pre-operative diagnosis : Malignant tumor of sigmoid colon 25CM FAV
- Post-operative diagnosis : Ditto

Surgical Indication

- Tumor resection

Operation

- Robot Assisted Colectomy(L)

Finding

- 1. One 2x2 cm tumor located at distal S-colon
  2. There was no palpable liver metastasis, no visible peritoneal seeding.
  3. High ligation (+) above the left colic artery.
  4. No drain
  5. Surgiwrap +

Procedure

- Under GE, the patient was put in lithotomy position. 5 troca hole was made, the first was by open method. Pneumoperitonium was created. the robotic endoscope was inserted. Abdominal exploration was done and the findings were mentioned above. The patient position was adjusted to head-down and right tilting to let small bowels move to RUQ and the pelvis was well exposured. The IMA was identified, dissected and transected by hemolock. The IMV was done in the same way. The mobilization of S-colon was done with dissection via embryonic plane between Gerota fascia and meso-sigmoid colon. The lateral attachement of sigmoid colon was also divided. After fully mobilization of S-colon, the rectum was also dissected posteriorly in upper rectum level, Denovillier fascia anteriorly. The bilateral lateral ligment was divided. Transection of rectum at R-S junction was done by 45 mm endocutters X1. The speciemn was removed from midline wound . The 29 mm envil was inserted in proximal colon annd take back into abdominal cavity. The end to end colorectal anastomosis was done by double stappling technique. After air-leak test, 4 ports and was wound closed in layers. Throughout the whole course of operation, patient stood quite well.
- Estimated blood loss: minimum
- 器械數量: 正確
- 紗布數量: 正確

Image

- PAINT
  - abdomen

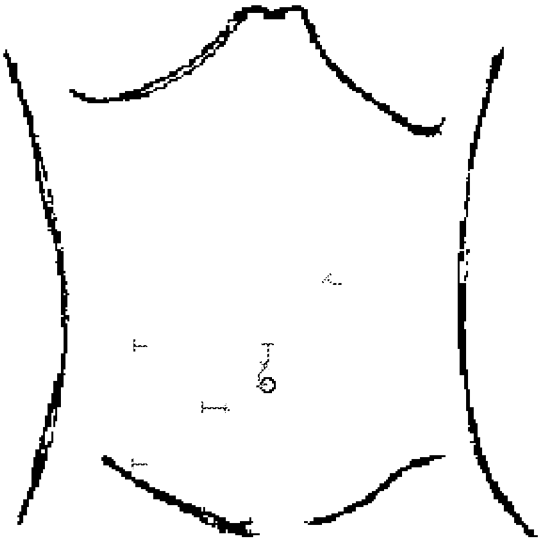

- colon & rectum

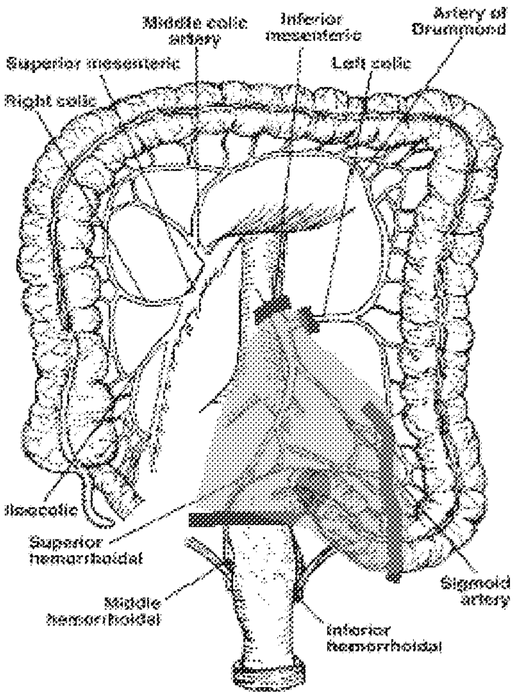

主任 2062F蔣鋒帆 / 醫師 2292G楊曙榮

正式摘要(**電子簽章：000000218421881)**

請選擇...reporter

doctorRank:B
